# Editorial: Recent breakthroughs in the decoding of circulating nucleic acids and their applications to human diseases

**DOI:** 10.3389/fmolb.2023.1203495

**Published:** 2023-05-10

**Authors:** Sherien M. El-Daly, Roba M. Talaat, Maria Braoudaki, Rana A. Youness, William C. Cho

**Affiliations:** ^1^ Medical Biochemistry Department, Medical Research and Clinical Studies Institute, National Research Centre, Giza, Egypt; ^2^ Molecular Biology Department, Genetic Engineering and Biotechnology Research Institute (GEBRI), University of Sadat City, El Sadat City, Egypt; ^3^ Clinical, Pharmaceutical, and Biological Science Department, School of Life and Medical Sciences, University of Hertfordshire, Hatfield, United Kingdom; ^4^ Biology and Biochemistry Department, School of Life and Medical Sciences, University of Hertfordshire Hosted by Global Academic Foundation, Cairo, Egypt; ^5^ Department of Clinical Oncology, Queen Elizabeth Hospital, Kowloon, Hong Kong SAR, China

**Keywords:** circulating nucleic acids, precision medicine, liquid biopsy, cell-free DNA, non-coding RNAs

The research on circulating nucleic acids (CNAs) is one of the most intriguing and rapidly growing research fields in recent years. The history of CNAs dates back to the 1940s (Mandel and Metais, 1948), and since then, numerous research has been conducted to investigate their clinical significance in various disorders. Research on CNAs has led to a wealth of information that enabled scientists to detect disease-specific genetic aberrations from extracellular fluids. Because of its significant diagnostic, prognostic, and predictive features, this discipline has lately emerged as the most recent development in precision medicine (Lone et al., 2022). Circulating nucleic acids signature covers several types of DNA and RNA molecules: cell-free DNA (cfDNA), circulating tumor DNA (ctDNA), microRNAs (miRNAs), long non-coding RNAs (lncRNAs), circular RNA (circRNA), and other types.

Despite recent efforts to employ CNAs as biomarkers, only a few have been approved for clinical use (Ignatiadis et al., 2021), highlighting the need for more clinical validation studies on their significance as biomarkers and their functional involvement in various pathways.

In this Research Topic, we aimed to cover recent advances in the implications of cell-free nucleic acids and their applications in human diseases. With numerous submissions, five peer-reviewed articles were accepted, and a total of 40 international researchers contributed.

Cell-free DNA (cfDNA) testing is the core of most liquid biopsy assays, with fragmentation pattern being one of the features. The clinical significance of cfDNA fragmentation was the focus of two original research articles. The study by Kumar et al. investigated the potential of cfDNA fragmentation patterns in differentiating hepatocellular carcinoma (HCC) from chronic liver disease (CLD). In this study, HCC patients and CLD patients with viral and non-viral etiologies, in addition to healthy individuals, were recruited. cfDNA was isolated from all participants, and fragments of repetitive elements (ALU and LINE1) and housekeeping genes (β-Actin and GAPDH) were measured. Total cfDNA concentrations and integrity index were also evaluated. Results showed that the total cfDNA concentrations in the sera of HCC patients were significantly higher than those of CLD patients and healthy individuals. Yet, HCC patients have shown poor DNA integrity or excess cfDNA fragmentation than CLD patients. The authors highlighted the increased cfDNA fragmentation trend among HCC patients of different etiologies, indicating that an elevated cfDNA fragmentation pattern is a hallmark of HCC progression regardless of HCC etiology.

Another study on cfDNA fragmentation was conducted on colorectal cancer (CRC) by Koval et al., who employed digital droplet PCR (ddPCR) to study cfDNA lengths using blood samples collected from healthy donors and previously untreated patients with CRC. They observed shorter and more variable cfDNA fragments across samples in open-chromatin regions relative to the pericentromeric closed chromatin locus. Meanwhile, they detected an increase in the relative fraction of shorter cfDNA fragments in CRC patients regardless of chromatin status, indicating that cfDNA shortening in cancer is not simply driven by cancer-related alterations in chromatin accessibility to nucleases. The authors also presented an efficient qPCR system suitable for screening cfDNA samples for artificial high molecular weight DNA contamination.

Circulating non-coding RNAs (ncRNAs) are promising, robust non-invasive biomarkers due to their high conservation, stability, and specificity (Mitchell et al., 2008; El-Daly et al., 2023). In a comprehensive umbrella review and pan-cancer analysis article, Bahramy et al. re-analyzed data from current systematic reviews and meta-analysis studies on the potential diagnostic and prognostic function of ncRNAs as biomarkers in breast cancer. Among various sources of ncRNAs, their analysis showed that blood-associated ncRNAs have better diagnostic values than tissue ncRNAs. Interestingly, the authors analyzed the diagnostic value of ncRNAs in Asian and Caucasian ethnicities, and the results were almost the same in these two ethnicities. According to their findings, miRNAs demonstrated a higher diagnostic accuracy than lncRNAs. Moreover, Combined miRNAs outperform single miRNAs as biomarkers for breast cancer detection.

On the other hand, Amer et al. studied the functional role of ncRNAs. The authors investigated the interlinkage between circulating MALAT1 and HOTAIR and their effects on the expression of oncogenic immunomodulatory proteins in tumor-associated macrophages (TAMs) of the HER2+ and triple-negative breast cancer (TNBC) subtypes. Their results showed a promising use of the lncRNAs (MALAT1 and HOTAIR) in regulating oncogenic immune-modulatory proteins MSLN and CD80 in TAMs of HER2+ and TNBC patients. Their findings shed light on novel key players affecting the anti-inflammatory activity of TAMs as a possible therapeutic target in HER2+ and TNBC patients.

In this Research Topic, we aimed to present the updated techniques used to characterize molecular features of cell-free nucleic acids associated with diseases. In an interesting work, Chatterjee et al. employed whole-exome sequencing (WES) on genomic DNA isolated from peripheral blood to uncover the underlying cause of kidney stones in lower age in West Bengal, India. Nephrolithiasis (NL), or kidney stone disease (KSD), is a common problem among the Indian population with multifactorial etiology that includes dietary, environmental, and genetic aspects (Guha et al., 2019). For the first time, Chatterjee et al. provided data on lower-age NL and its prevalence in West Bengal, India. The authors of this study performed whole exome sequencing on two age groups; 0–20 years as the lower age group (pediatric KSD) and above 20 years (adult KSD) group. The results showed the presence of 25 mutations in 18 genes from the pediatric KSD group that were missed in adults. In the progression of KSD, authors monitored the impact of the prevalent c.494G > A GRHPR mutation. Results revealed that the lower age group of patients is more likely to develop hyperoxaluria and have the GRHPR gene mutation c.494G > A, according to WES analysis. The upregulation of the gene with decreased enzyme activity due to the mutation was supported by functional and biochemical studies. This study emphasized the significance of GRHPR c.494G > A mutation as a clinical biomarker and monogenic cause of lower-age KSD in the West Bengal community.

Collectively, the publications of this Research Topic provide some contemporary studies of circulating nucleic acids, their biological functions, and their molecular diagnostic potentials. These insights could help pave the way for a new era in personalized medicine.
